# TRAJECTORIES OF MOTORIC COGNITIVE RISK SYNDROME AND INCIDENT DEMENTIA IN SCOTLAND

**DOI:** 10.1093/geroni/igad104.2761

**Published:** 2023-12-21

**Authors:** Donncha Mullin, Danni Gadd, Michelle Luciano, Tom Russ, Graciela Muniz-Terrera

**Affiliations:** University of Edinburgh, Edinburgh, Scotland, United Kingdom; University of Edinburgh, Edinburgh, Scotland, United Kingdom; University of Edinburgh, Edinburgh, Scotland, United Kingdom; University of Edinburgh, Edinburgh, Scotland, United Kingdom; Ohio University, Athens, Ohio, United States

## Abstract

**Background:**

Motoric cognitive risk syndrome (MCR) - objective slow gait and subjective cognitive complaints - is quick and easy to measure, making it a potentially useful clinical tool for identifying those at high risk of developing dementia. This is the first exploration of MCR syndrome’s predictive ability for incident dementia in a Scottish cohort (Lothian Birth Cohort 1936) and the first examination of the various trajectories of MCR. Method: We classified a total of 680 community-dwelling participants (mean [SD] age 76.3 [0.8] years) free from dementia into non-MCR or MCR groups. We used cox proportional hazard methods to evaluate the risk of developing all-cause dementia in the years following MCR diagnosis, adjusting for age, sex, comorbidity index, apolipoprotein E status, physical activity level, and years of education. We analysed the trajectories for everyone with MCR by tracking their outcomes over 9 years. Result: The prevalence of MCR at baseline was 5.6% (n=38/680). After 9 years of follow-up, 11.6% (n=79/680) of the total cohort developed dementia. The presence of MCR increased the risk of dementia (adjusted hazard ratio 2.37 [95%CI 1.06-5.32; p=0.0359]). The trajectories of those with MCR at baseline were: i) reverted to healthy (n=6/38), ii) ongoing MCR (n=13/38), iii) progressed to dementia (n=9/38), or iv) died (n=10/38).

**Conclusion:**

MCR could identify a target group for early interventions of modifiable risk factors to prevent incident dementia. Individuals with MCR follow similar trajectories to the related predementia syndrome, Mild Cognitive Impairment.

